# TKT maintains intestinal ATP production and inhibits apoptosis-induced colitis

**DOI:** 10.1038/s41419-021-04142-4

**Published:** 2021-09-17

**Authors:** Na Tian, Lei Hu, Ying Lu, Lingfeng Tong, Ming Feng, Qi Liu, Yakui Li, Yemin Zhu, Lifang Wu, Yingning Ji, Ping Zhang, Tianle Xu, Xuemei Tong

**Affiliations:** 1 Department of Neurology, Shandong Provincial Hospital Affiliated to Shandong First Medical University, Jinan, Shandong China; 2 Department of Biochemistry and Molecular Cell Biology, Shanghai Key Laboratory for Tumor Microenvironment and Inflammation, Key Laboratory of Cell Differentiation and Apoptosis of Chinese Ministry of Education, Shanghai Jiao Tong University School of Medicine, Shanghai, China; 3 Department of Physiology , Weifang Medical University, Weifang, Shandong China; 4 Department of Anatomy and Physiology, Center for Brain Science of Shanghai Children’s Medical Center, Shanghai Jiao Tong University School of Medicine, Shanghai, China

**Keywords:** Apoptosis, Mechanisms of disease, Inflammatory bowel disease

## Abstract

Inflammatory bowel disease (IBD) has a close association with transketolase (TKT) that links glycolysis and the pentose phosphate pathway (PPP). However, how TKT functions in the intestinal epithelium remains to be elucidated. To address this question, we specifically delete TKT in intestinal epithelial cells (IECs). IEC TKT-deficient mice are growth retarded and suffer from spontaneous colitis. TKT ablation brings about striking alterations of the intestine, including extensive mucosal erosion, aberrant tight junctions, impaired barrier function, and increased inflammatory cell infiltration. Mechanistically, TKT deficiency significantly accumulates PPP metabolites and decreases glycolytic metabolites, thereby reducing ATP production, which results in excessive apoptosis and defective intestinal barrier. Therefore, our data demonstrate that TKT serves as an essential guardian of intestinal integrity and barrier function as well as a potential therapeutic target for intestinal disorders.

## Introduction

The mammalian intestinal epithelium is composed of a monolayer of columnar epithelial cells organized into crypts and villi that renews every 4–5 days [1]. It is an important metabolic tissue, serving a variety of physiological functions including digestion and absorption of nutrients as well as forming a physical and biochemical barrier against enteric pathogens to maintain tissue homeostasis [1–3]. Once the intestinal barrier function is disturbed by various risk factors such as deregulated apoptosis, the intestinal mucosa directly contacts with the luminal invading pathogens and ingested toxins to promote inflammatory responses, leading to intestinal disorders including inflammatory bowel disease (IBD) [4].

Recent studies have intensively suggested a strong link between intestinal disorders and metabolites or metabolic genes. Some products from carbon metabolism, such as pyruvate, play an anti-inflammatory role in colitis [5, 6]. In addition, lipids, short fatty acids and amino acids also have a close association with intestinal barrier function and IBD [7–10]. Moreover, metabolic genes including pyruvate kinase (PKM2), NADPH oxidase (NOX) and TIGAR have been shown to involve in the progression of IBD [11–13]. Thus, focus on the regulation of metabolism in IBD may provide new insights into clinical therapies.

Transketolase (TKT), linking the pentose phosphate pathway (PPP) and glycolysis, plays a critical role in the non-oxidative PPP [14, 15]. TKT catalyzes two reversible reactions, determining the direction of metabolites in PPP according to metabolic demands [15]. One reaction catalyzed by TKT is the conversion of xylulose-5-phosphate (Xu5P) and ribose-5-phosphate (R5P) into glyceraldehyde-3-phosphate (G3P) and sedoheptulose-7-phosphate (S7P). The other is to produce G3P and fructose-6-phosphate (F6P) from Xu5P and erythrose-4-phosphate (E4P) [15]. Our previous data have shown that TKT serves as a critical regulator of carbohydrate and lipid catabolism [16]. Cellular TKT deficiency accumulates non-oxidative PPP metabolites and decreases glycolytic products [16, 17], which are associated with intestinal barrier function [5, 6, 18]. The TKT activity is dependent on thiamine, and thiamine deficiency has a strong association with IBD [19]. Moreover, erythrocyte TKT activity is declined in patients suffering from IBD [20]. Furthermore, lack of transketolase-like 1 (TKTL1), which belongs to the TKT gene family, aggravates murine experimental colitis [21]. However, the functions of TKT gene family in regulating intestinal homeostasis remain unknown.

In mice, inactivation of both TKT alleles is lethal whereas disruption one of TKT alleles causes growth retardation and preferential reduction of adipose tissues [22]. Nevertheless, our previous data have indicated that TKT ablation in the liver and adipose tissues has no effect on the growth and development in mice fed with normal chow diet [16, 17]. Therefore, TKT downregulation in other tissues such as the intestine possibly accounts for the growth retardation phenotypes.

To investigate the possible role of TKT in intestinal physiology, we generate an intestinal epithelium-specific TKT knockout mouse model. In the present study, we show that TKT deficiency in the intestinal epithelium results in reduction of glycolytic metabolites and insufficient ATP production, which can lead to epithelial cell apoptosis, intestinal barrier defects, spontaneous colitis and mouse growth retardation. Therefore, TKT maintains intestinal homeostasis through protecting epithelial cells from energy insufficiency and apoptosis.

## Results

### TKT deletion in intestinal epithelium leads to growth retardation in mice

**Fig. 1:**
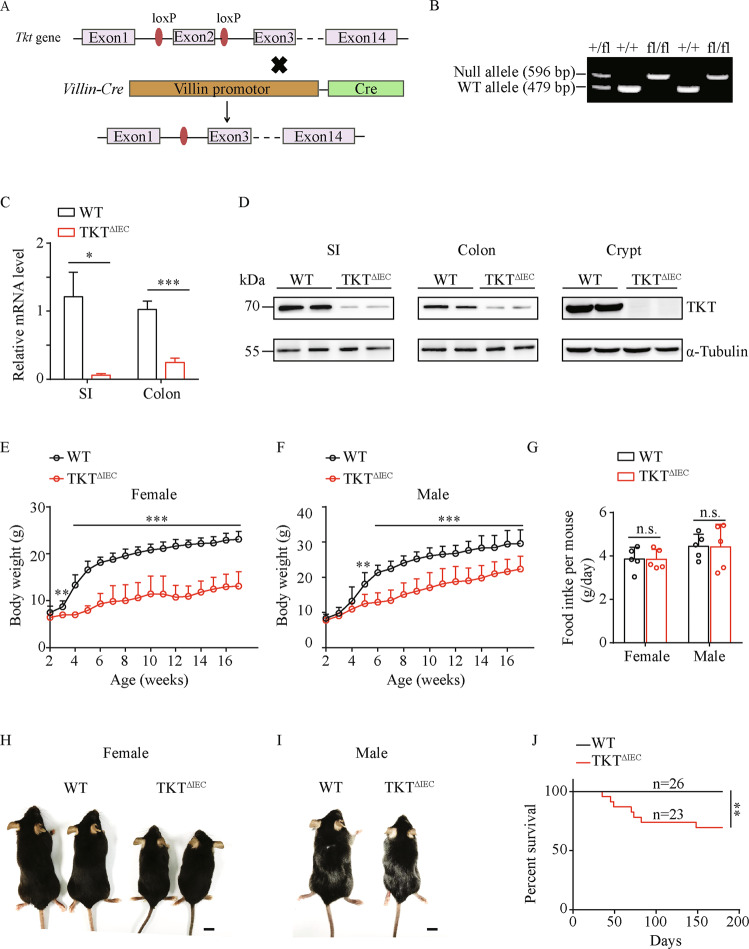
TKT deficiency in intestinal epithelium results in growth retardation. **A** Schematic diagram of the strategy to generate TKT^∆IEC^ mice. **B** PCR analysis for TKT WT and null alleles. **C** QPCR analysis of TKT mRNA expression in small intestines and colons from WT and TKT^∆IEC^ mice (*n* = 5). **D** Western blotting analysis of TKT protein levels in tissues from WT and TKT^∆IEC^ mice. **E** Body weight of female WT and TKT^∆IEC^ mice at different ages (*n* = 9 for WT mice, *n* = 8 for TKT^∆IEC^ mice). **F** Body weight of male WT and TKT^∆IEC^ mice at different ages (*n* = 8 for WT mice, *n* = 10 for TKT^∆IEC^ mice). **G** Food intake of WT and TKT^∆IEC^ mice (*n* = 5). **H** Representative images of female WT and TKT^∆IEC^ mice at the age of 4 months. Scale bars, 1 cm. **I** Representative images of male WT and TKT^∆IEC^ mice at the age of 3 months. Scale bars, 1 cm. **J** Kaplan–Meier survival curve of WT and TKT^∆IEC^ mice (log-rank test). Data are represented as mean ± SEM. Significance is determined by Student’s *t* test analysis, *P < 0.05, **P < 0.01, ***P < 0.001.

To directly investigate the physiological functions of TKT in the intestine, we generated IEC-specific TKT knockout (abbreviated as TKT^ΔIEC^) mice by crossing *Tkt*^*fl/fl*^ mice with *Villin-Cre* mice (Fig. 1A). Polymerase chain reaction (PCR) was performed to identify different genotypes (Fig. 1B). TKT expression was notably decreased in the small intestine (SI), colon and crypts in TKT^ΔIEC^ mice compared with WT mice (Fig. 1C, D).

TKT^ΔIEC^ mice were born at a Mendelian ratio and became distinguishable from their WT littermates due to significant growth retardation from 3 weeks old. Without difference in food intake, both female and male TKT^ΔIEC^ mice showed lower body weight and smaller size after weaning, which became more significant as the mice aged (Fig. 1E–I). Moreover, a small percentage of TKT^ΔIEC^ mice died at different ages (Fig. 1J). Collectively, these data suggest that sufficient TKT in IECs is critical for murine normal growth.

### TKT^ΔIEC^ mice spontaneously develop colitis

**Fig. 2:**
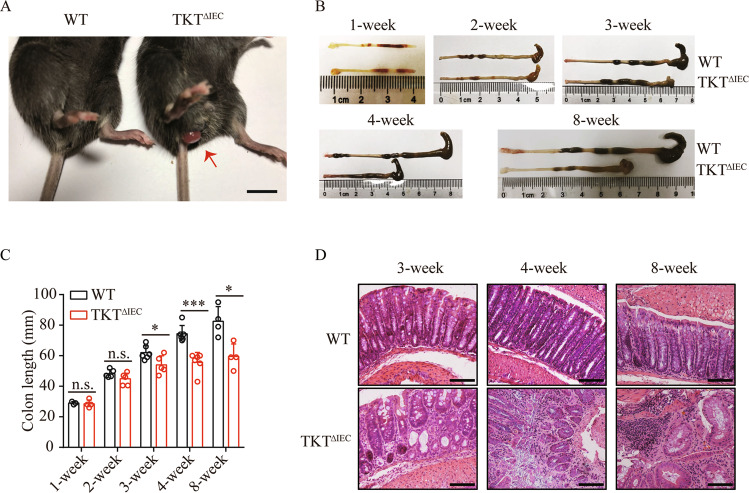
Loss of TKT in intestinal epithelium induces spontaneous colitis. **A** TKT^∆IEC^ mice are more susceptible to rectocele compared with their WT littermates. Scale bars, 1 cm. **B** Representative images of colons of WT and TKT^∆IEC^ mice at different ages. **C** Quantification of colon length of WT and TKT^∆IEC^ mice at different ages. **D** Representative H&E-stained colon sections of WT and TKT^∆IEC^ mice at different ages. Scale bars, 200 μm.

When exploring how loss of TKT in IECs resulted in growth retardation, we observed that TKT^ΔIEC^ mice displayed symptoms of colitis, including frequent loose stools, diarrhea and even rectal prolapses (Fig. 2A). Notably, the colons of TKT^ΔIEC^ mice were much shorter than those of WT mice starting from 3 weeks of age, often accompanied by abnormal cecum (Fig. 2B, C). Histological analysis revealed that TKT deficiency altered the colonic morphology, with dilated crypt diameters, oval or slit-like lumens and thickened epithelial linings (Fig. 2D). As the mice aged, TKT^ΔIEC^ animals showed increased epithelial disruption, follicle aggregation, crypt loss, enhanced erosion and even aberrant crypt foci (ACF), compared to WT littermates (Fig. 2D).

**Fig. 3:**
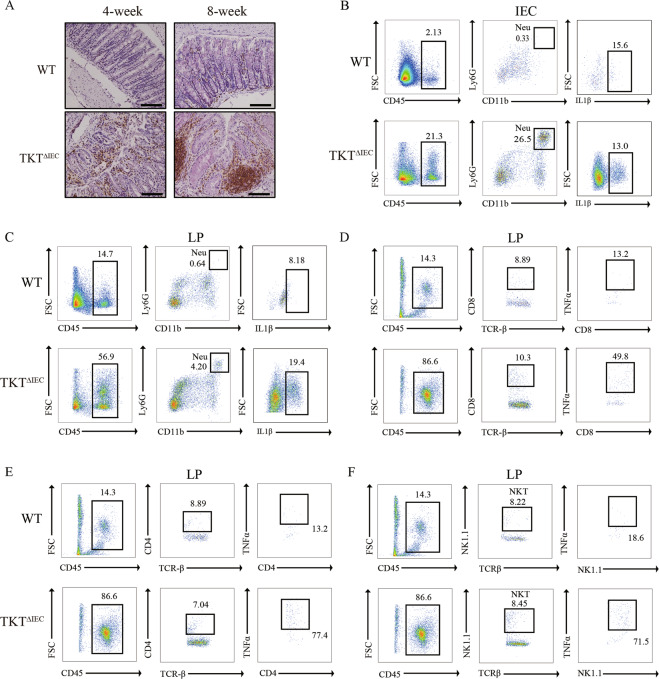
TKT ablation in intestinal epithelium leads to massive infiltration of immune cells. **A** Representative images of colon sections stained with the CD45 antibody from WT and TKT^∆IEC^ mice at different ages. Scale bars, 100 μm. **B** Representative flow cytometric analysis of IL-1β from neutrophils in the IEC of colons from 6-week-old WT and TKT^∆IEC^ mice (*n* = 3). **C** Representative flow cytometric analysis of IL-1β from neutrophils in the LP of colons from 6-week-old WT and TKT^∆IEC^ mice (*n* = 3). **D** Representative flow cytometric analysis of TNFα from CD8^+^ T cells in the LP of colons from 6-week-old WT and TKT^∆IEC^ mice (*n* = 3). **E** Representative flow cytometric analysis of TNFα from CD4^+^ T cells in the LP of colons from 6-week-old WT and TKT^∆IEC^ mice (*n* = 3). **F** Representative flow cytometric analysis of TNFα from NKT cells in the LP of colons from 6-week-old WT and TKT^∆IEC^ mice (*n* = 3).

Moreover, TKT deficiency resulted in immune cell infiltration in both epithelial and lamina propria fractions and bigger Peyer’s patch (Fig. 3A). Flow cytometry analysis confirmed increased immune cell infiltration in intestinal epithelial cell (IEC) and lamina propria (LP) of TKT^∆IEC^ colons (Fig. 3B, C). Among different subsets of immune cells infiltrated in colon tissues, neutrophils were the most significantly increased population in both IEC and LP of TKT^∆IEC^ colons when compared with WT (Fig. 3B, C, Supplementary Fig. S1). T cells were slightly increased in TKT^∆IEC^ mice whereas B cells, macrophages and dendritic cells remained unchanged between WT and TKT^∆IEC^ mice (Supplementary Fig. S1). Furthermore, levels of IL-1β produced by neutrophils in both IEC and LP as well as TNFα produced by CD8^+^ T cells, CD4^+^ T cells and natural killer T (NKT) cells in LP of TKT^∆IEC^ mice were also elevated (Fig. 3C–F). In addition, quantitative PCR (qPCR) analysis showed that IL-1α, IL-1β, IL-6, TNFα and Cox-2 were all transcriptionally upregulated in colonic tissues from TKT^ΔIEC^ mice in comparison with WT controls (Supplementary Fig. S2A). Consistently, the circulating levels of pro-inflammatory cytokines, including IL-6 and TNFα, were significantly higher in serum from TKT^ΔIEC^ mice (Supplementary Fig. S2B, C). Together, these results demonstrate that TKT ablation leads to intestinal inflammation with extensive epithelial erosion and inflammatory cell infiltration.

### Intestinal epithelial TKT ablation causes defective barrier function

**Fig. 4:**
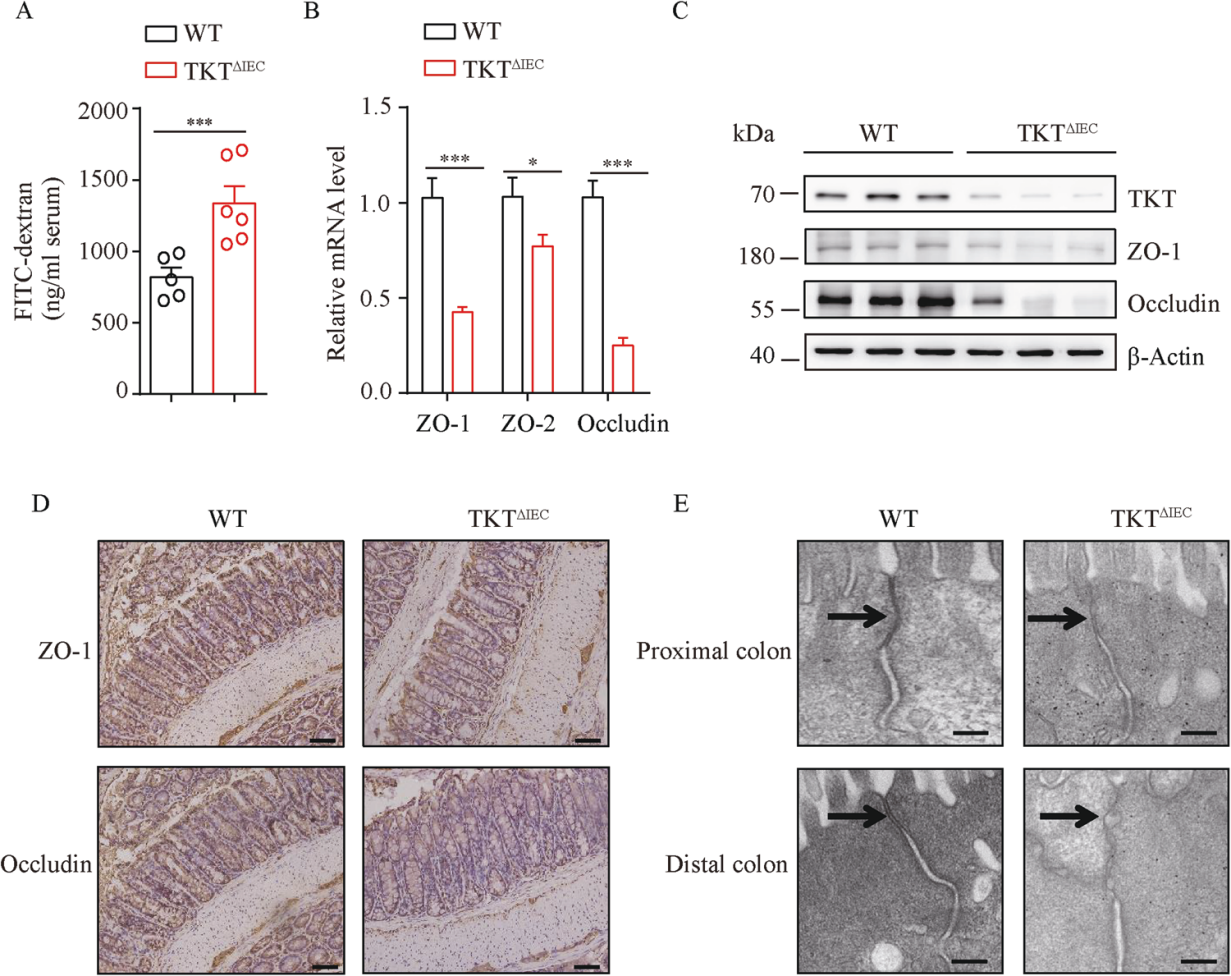
IEC TKT deletion disturbs barrier function of intestines. **A** Intestinal permeability was measured by determining the concentration of FITC-dextran in blood serum (*n* = 5 for WT mice, *n* = 6 for TKT^∆IEC^ mice). **B** QPCR analysis of the expression of genes related to tight junction in colons (*n* = 7 for WT mice, *n* = 9 for TKT^∆IEC^ mice) from 8-week-old WT and TKT^∆IEC^ mice. **C** Western blotting analysis of proteins in colon lysates from 3-week-old WT and TKT^∆IEC^ mice (*n* = 3). **D** Representative images of colon sections stained with the indicated antibodies from 8-week-old WT and TKT^∆IEC^ mice. Scale bars, 50 μm. **E** Electron micrographs of intestinal epithelial cells from proximal colon (top row) and distal colon (bottom row) of WT and TKT^∆IEC^ mice showing the tight junction structures. Arrows point to tight junction membrane kisses which were disrupted in TKT^∆IEC^ mice. Scale bars, 200 nm. Data are represented as mean ± SEM. Significance is determined by Student’s *t* test analysis, *P < 0.05, **P < 0.01, ***P < 0.001.

Epithelial tight junctions have a strong link with colitis, through regulating the paracellular permeability and the integrity of the epithelial barrier [23, 24]. Therefore, we analyzed the effect of TKT deletion on epithelial tight junctions and intestinal permeability. Using fluorescein isothiocyanate (FITC)-dextran [25], we found that in vivo intestinal permeability was notably increased in TKT^ΔIEC^ mice (Fig. 4A). In line with this, expression of key components of tight junctions including zonula occludens-1 (ZO-1), ZO-2 and Occludin [24], was notably decreased in the colons of TKT^ΔIEC^ mice in comparison with WT mice (Fig. 4B–D). To further examine the effect of TKT deficiency on the morphology of tight junctions in epithelial cells of colons, we compared the ultrastructure of tight junctions in WT and TKT^ΔIEC^ colons by electron microscopy. Intestinal epithelial cells from WT mice showed the characteristic tight junction membrane kisses (Fig. 4E). However, loss of TKT resulted in an apparent deformation of tight junctions in epithelial cells of colons (Fig. 4E). These data indicate that TKT plays an important role in regulating intestinal epithelial tight junction assembly and barrier function.

### Loss of TKT in IECs induces apoptosis and ectopic proliferation

**Fig. 5:**
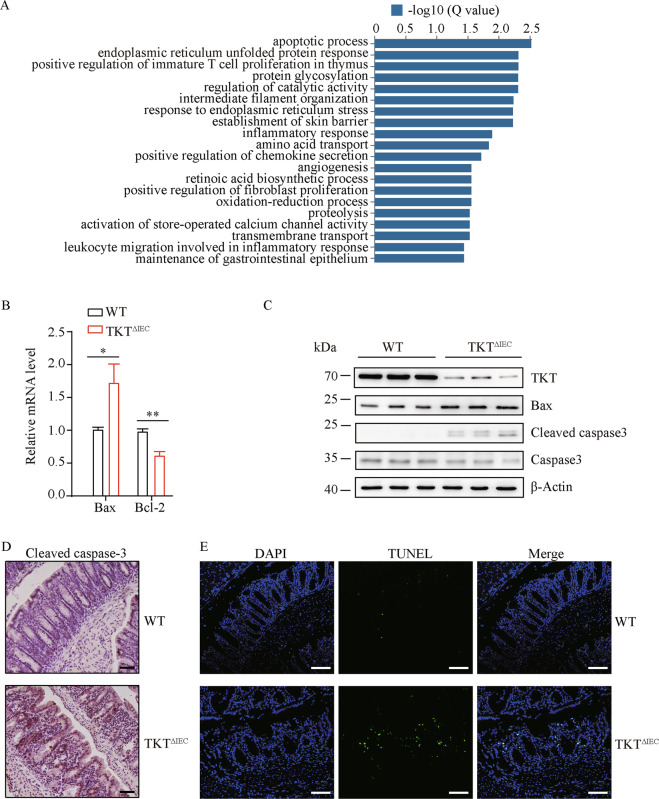
TKT abrogation leads to apoptosis in intestinal epithelium. **A** Gene ontology analysis of upregulated genes in colons of 4-week-old WT and TKT^∆IEC^ mice. **B** QPCR analysis of mRNA levels of apoptotic genes in colons from 3-week-old WT and TKT^∆IEC^ mice (*n* = 5). **C** Colon lysates were analyzed by western blotting (one mouse per lane) with the indicated antibodies from 3-week-old WT and TKT^∆IEC^ mice. **D** Representative images of colon sections stained with the cleaved caspase-3 antibody from 3-week-old WT and TKT^∆IEC^ mice. Scale bars, 50 μm. **E** Representative images of TUNEL-stained colon sections of 3-week-old WT and TKT^∆IEC^ mice. Scale bars, 75 μm. Data are represented as mean ± SEM. Significance is determined by Student’s *t* test analysis, *P < 0.05, **P < 0.01, ***P < 0.001.

To further investigate the mechanism underlying TKT deficiency-induced colitis, we performed RNA sequencing (RNA-seq) using the colonic tissues of 4-week-old WT and TKT^∆IEC^ mice. The Gene Ontology (GO) enrichment analysis of RNA-seq data showed that genes involved in the apoptotic process were most significantly changed among the upregulated genes (Fig. 5A). Excessive IEC apoptosis is a well-recognized mechanism of colitis by causing severe gut pathology with mucosal damage and leukocyte infiltration [4, 26–28]. Apoptosis is a complex process in which the pro-apoptotic protein Bax and the anti-apoptotic protein Bcl-2 play critical roles [29, 30]. Notably, Bax was significantly upregulated, whereas Bcl-2 was downregulated in colons of 3-week-old TKT^∆IEC^ mice compared to WT mice (Fig. 5B, C). Caspase-3, a main executor of the apoptotic process, is often markedly elevated in colonic tissues of colitis [27, 31]. Consistently, we found that TKT deletion upregulated the colonic expression of cleaved caspase-3 (Fig. 5C, D). In addition, TUNEL staining showed more apoptotic epithelial cells in TKT^∆IEC^ colonic tissues compared to WT controls (Fig. 5E).

**Fig. 6:**
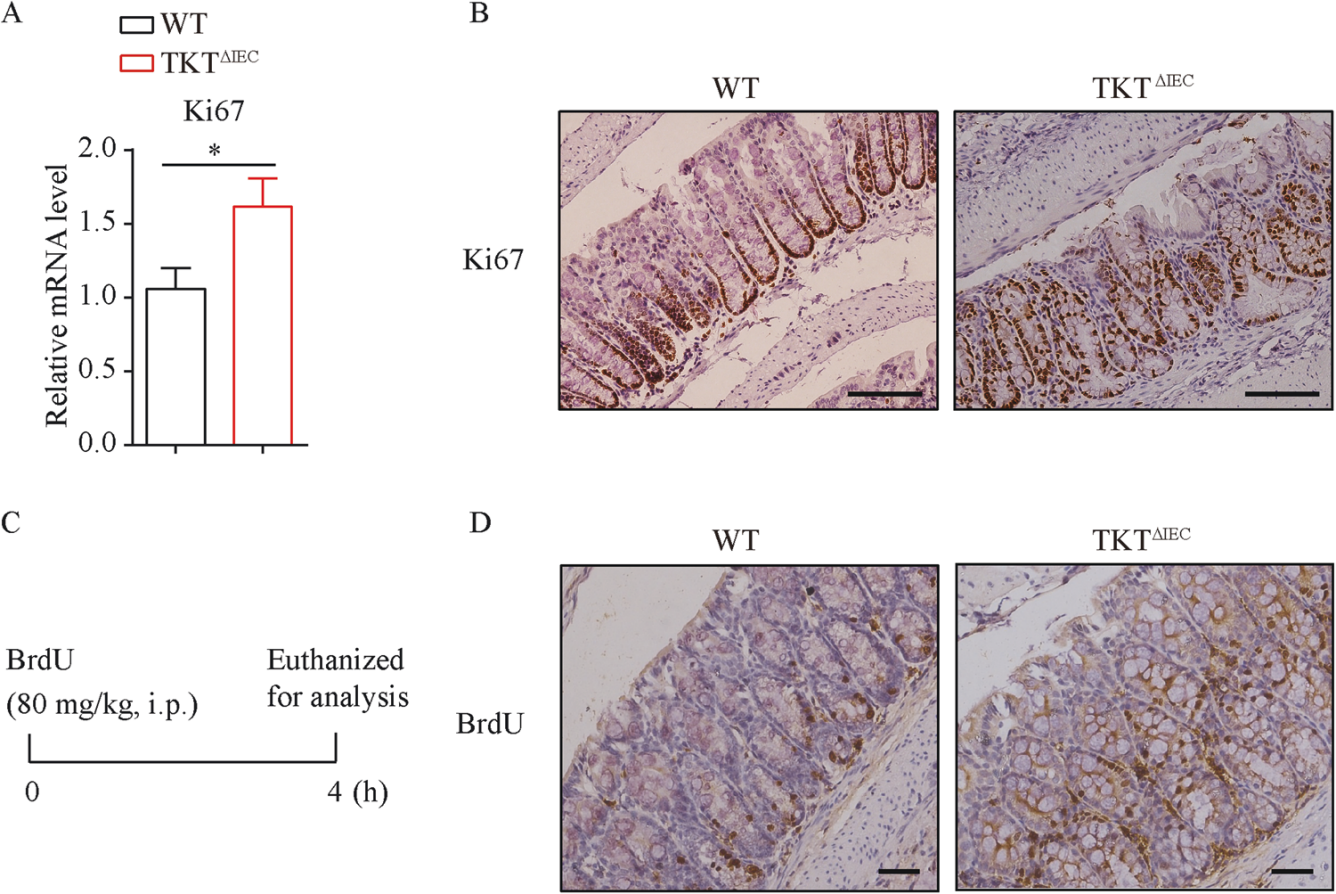
TKT abrogation induces ectopic hyperproliferation in intestinal epithelium. **A** QPCR analysis of Ki67 expression in colons from 8-week-old WT and TKT^∆IEC^ mice (*n* = 7 for WT mice, *n* = 9 for TKT^∆IEC^ mice). **B** Representative images of colon sections stained with the Ki67 antibody from 8-week-old WT and TKT^∆IEC^ mice. Scale bars, 100 μm. **C** Schematic procedure of the BrdU pulse-chase experiment. **D** Representative images of colon sections stained with the BrdU antibody from 8-week-old WT and TKT^∆IEC^ mice i.p. injected with BrdU 4 h before euthanization. Scale bars, 50 μm. Data are represented as mean ± SEM. Significance is determined by Student’s *t* test analysis, *P < 0.05, **P < 0.01, ***P < 0.001.

Continuous self-renewal of the intestinal epithelium critically depends on proliferation of stem cells within the crypts and apoptosis at the villous tip [4, 32]. Epithelial cell proliferation and apoptosis needs to be tightly regulated. During the self-renewable process, proliferation is restricted to cells at the bottom of crypts, whereas apoptosis generally occurs on superficial cells lining the lumen [4]. Therefore, we next investigated the effects of excessive apoptosis due to TKT deficiency on intestinal epithelial proliferation. QPCR analysis showed a significant increase in Ki67 expression, suggesting that TKT^∆IEC^ mice had more proliferative cells (Fig. 6A). Moreover, IHC staining showed that Ki67 protein was dispersed throughout the crypt of TKT^∆IEC^ mice rather than at the base of the crypt, where the proliferative cells should reside as shown in controls (Fig. 6B). Furthermore, a BrdU pulse-chase experiment also showed that BrdU-stained cells were distributed throughout the entire crypt of TKT^∆IEC^ mice instead of being concentrated near the base of the crypt in WT (Fig. 6C, D).

Collectively, these data reveal that TKT deficiency disturbs the balance between intestinal epithelial apoptosis and proliferation, resulting in excessive apoptosis and ectopic hyperproliferation.

### TKT deficiency in the intestinal epithelium limits energy supply

**Fig. 7:**
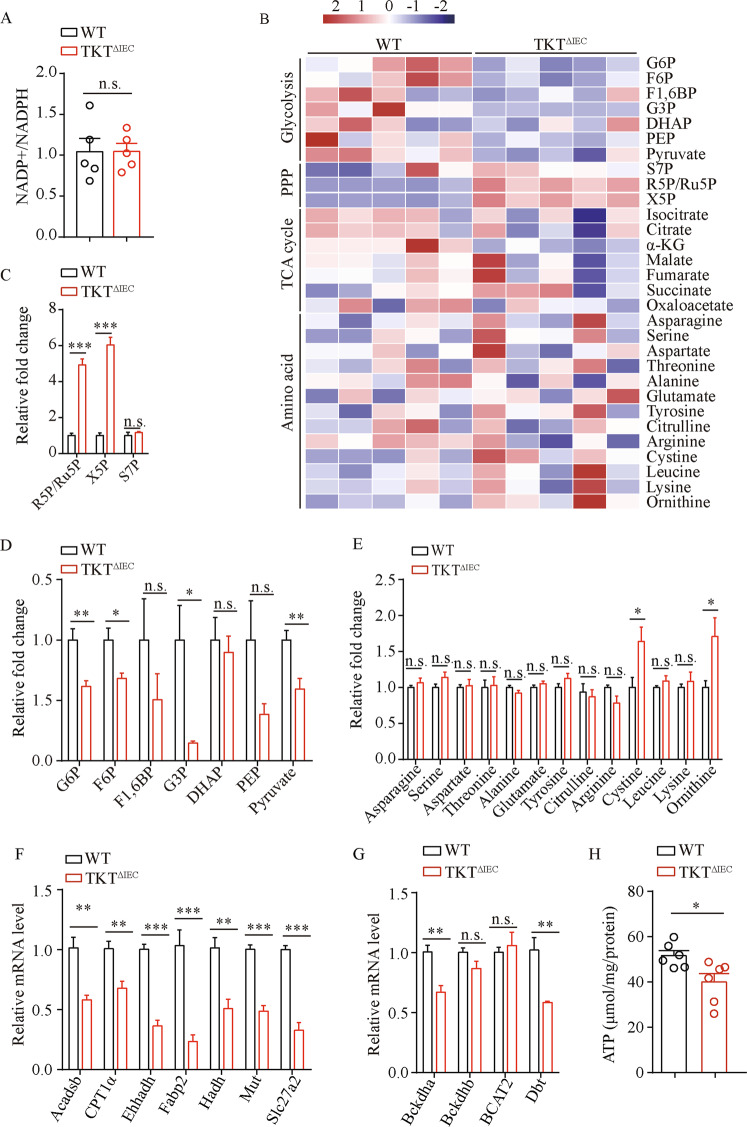
Loss of TKT causes insufficient energy supply in the intestine. **A** NADP^+^/NADPH levels in colons of 3-week-old WT and TKT^∆IEC^ mice (*n* = 5). **B** Heat map of metabolites in glycolysis, PPP, TCA cycle and amino acids in colons of 4-week-old WT and TKT^∆IEC^ mice. **C** Relative levels of PPP metabolites in colons of 4-week-old WT and TKT^∆IEC^ mice (*n* = 5). **D** Relative levels of glycolytic metabolites in colons of 4-week-old WT and TKT^∆IEC^ mice (*n* = 5). **E** Relative levels of amino acids in colons of 4-week-old WT and TKT^∆IEC^ mice (*n* = 5). **F** QPCR analysis of mRNA levels of FAO-related genes in colons of 3-week-old WT and TKT^∆IEC^ mice (*n* = 5). **G** QPCR analysis of mRNA levels of enzyme genes for BCAA catabolism in colons of 3-week-old WT and TKT^∆IEC^ mice (*n* = 5). **H** Levels of colonic ATP of 3-week-old WT and TKT^∆IEC^ mice (*n* = 6). Data are represented as mean ± SEM. Significance is determined by Student’s *t* test analysis, *P < 0.05, **P < 0.01, ***P < 0.001.

Oxidative stress has been shown to play an important role in the apoptotic process [33]. PPP makes a great contribution to intracellular redox homeostasis by regulating NADPH generation [15]. Our previous studies have suggested that cellular TKT loss disrupts redox homeostasis by reducing NADPH and GSH levels and increasing reactive oxygen species (ROS) production [16, 17]. Therefore, we investigated whether TKT abrogation affected redox status in the intestine. Unexpectedly, there was no difference in colonic NADPH level between TKT^∆IEC^ and WT mice (Fig. 7A). Therefore, apoptosis induced by TKT deficiency in intestinal epithelium is not due to NADPH alteration or oxidative stress.

In addition to ROS, cellular energy crisis is also an important inducer of apoptosis [34]. We have found that TKT modulates glucose and lipid metabolism to maintain energy balance in adipose tissues [16]. Accordingly, we investigated whether TKT deletion altered intestinal energy metabolism. To this end, we first performed targeted detection of energy metabolomics using colonic tissues from 4-week-old TKT^∆IEC^ and WT mice. TKT deficiency led to accumulation of R5P, ribulose-5-phosphate (Ru5P) and Xu5P in the non-oxidative PPP, while levels of glycolytic metabolites were significantly reduced (Fig. 7B–D), consistent with our previous findings [16, 17]. Glucose is the major carbon source for cellular energy generation [35]. Thus, the reduction of glucose catabolism without compensatory fuel utilization usually suppresses ATP production. In adipose tissues, TKT deficiency causes significantly decreased glycolysis and a compensatory increase of lipid and branched amino acid (BCAA) catabolism to meet energy demands [16]. However, levels of amino acids in TKT-deficient colonic tissues remained mostly comparable with and even higher than those in controls (Fig. 7E). Moreover, the expression of enzyme genes involved in fatty acid oxidation (FAO) and BCAA catabolism was notably decreased in TKT-deficient colonic tissues (Fig. 7F, G), which is different from our findings in adipose tissues [16]. As a result, ATP production was significantly decreased in colonic tissues of TKT^∆IEC^ mice in comparison with WT controls (Fig. 7H), which probably led to intestinal epithelial apoptosis.

## Discussion

Our work has identified an unexpected role of TKT in maintaining the energy status and integrity of the intestine. TKT^ΔIEC^ mice spontaneously developed colitis, showing similar phenotypes to IBD [36]. Interestingly, loss of TKT in intestinal epithelium leads to excessive apoptosis and defective barrier function, which is probably due to insufficient energy supply. Therefore, our work has demonstrated that TKT-mediated glucose catabolism is indispensable for intestinal cell survival and barrier function.

Extensive evidences have indicated that excessive apoptosis of IECs contributes to the development of IBD by altering the structural integrity, amplifying the mucosal immune responses and perpetuating chronic intestinal inflammation [26, 27, 31, 37]. Therefore, regulation of apoptosis of IECs facilitates the intestinal homeostasis. Consistently, in the present study, we discovered that TKT deficiency aggravated intestinal epithelial apoptosis (Fig. 5). Since the intestinal epithelium is self-renewed, the maintenance of intestinal barrier requires a delicate and dynamic balance between epithelial loss by apoptosis and new cell generation by proliferation [4]. Nevertheless, although TKT deletion indeed caused compensatory proliferation (Fig. 6A), which might be due to R5P accumulation (Fig. 7B, C), the hyperproliferation was ectopic and deregulated (Fig. 6B, D). Thus, the balance between apoptosis and proliferation was disrupted, leading to defects in tight junction and barrier (Fig. 4). Furthermore, the ectopic proliferation probably contributes to the progression of ACF in older TKT^ΔIEC^ mice (Fig. 2D).

It has been reported that ROS can induce apoptosis and intestinal epithelial damage [38]. Although ROS is increased in TKT-deficient liver and adipose tissues due to the reduction of NADPH and GSH according to our previous data [16, 17], TKT ablation did not alter the level of NADP^+^/NADPH in the colonic tissues (Fig. 7A). Moreover, administration of antioxidants N-acetyl-cysteine (NAC) in drinking water failed to alleviate symptoms in TKT^ΔIEC^ mice (data not shown). Given the special role of intestinal microbiota in regulating NADPH production [39], one possible explanation is that the intestine may have more diverse ways to maintain NADP^+^/NADPH.

Apart from ROS, cellular ATP level is also an important determinant for apoptosis. As a tightly regulated process, apoptosis is an energy-requiring process, in which a variety of ATP-dependent steps are involved, such as caspase activation, enzymatic hydrolysis of macromolecules, chromatin condensation, and apoptotic body formation [34, 40–42]. On the other hand, when ATP falls below a certain level while there is still enough ATP for apoptotic process, apoptosis ensues. However, when there is a severe drop in cellular ATP, necrosis occurs [34, 41, 43]. Accordingly, here in our study, loss of TKT gave rise to a moderate decrease of ATP in the intestinal epithelium (Fig. 7H), probably accounting for the increase of epithelial apoptosis.

Glucose, a major source of ATP for multicellular organisms, can be metabolized by both glycolysis and PPP. Our previous findings have shown that TKT inactivation disturbs the crosstalk between the two pathways, causing accumulation of PPP metabolites and reduction of glycolytic metabolites [16, 17], which indicates a decrease of glucose-derived ATP production. Consistently, TKT deletion reduced the metabolite levels of glycolysis in the intestinal epithelium (Fig. 7B, D). However, in contrast to adipose tissues, TKT deficiency in intestines downregulated the enzymatic genes involved in FAO and BCAA catabolism (Fig. 7F, G). As a result, intestinal epithelial ATP declined due to TKT loss (Fig. 7H), which induced apoptosis. It will be worthwhile to figure out why other fuels such as fatty acids and amino acids fail to compensate for reduced glucose catabolism in the future.

**Fig. 8:**
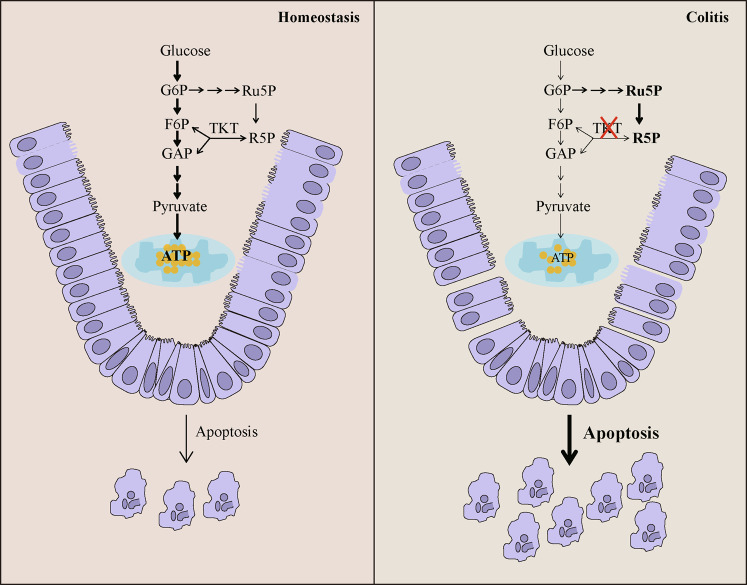
Graphical model. TKT serves as a safeguard to maintain intestinal homeostasis and barrier function by regulating ATP production and apoptosis.

In summary, our study has demonstrated for the first time that, TKT serves as a safeguard to maintain intestinal homeostasis and barrier function by regulating ATP production and apoptosis (Fig. 8). These findings may provide new insights into the physiology and pathology of the gut as well as designing therapeutic targets for clinical intestinal disorders.

## Materials and methods

### Animals

Intestinal epithelium-specific TKT knockout mice were generated by crossing *Tkt*^*fl/fl*^ mice [17] and *Villin-Cre* mice (Shanghai Biomodel Organism Science & Technology Development Co., Ltd.). All mice were in C57BL/6J background. Mice were housed in a temperature-controlled environment at constant room temperature (22 °C) under a 12 h light/dark cycle with free access to water and food. All animal experiments were approved by the Shanghai Jiao Tong University School of Medicine Animal Care and Use Committee.

### Isolation of intestinal crypts

Crypts were isolated as previously described [44]. Briefly, the fresh large intestine without cecum was isolated, dissected, opened longitudinally, and cut into 1 cm pieces. Next, these pieces were washed thoroughly with ice-cold PBS three times and further chopped into 5 mm pieces, followed by incubation in 5 mM EDTA/PBS at 4 °C on a rocking platform for 30 min. Crypts were released by shaking the tubes for 2 min and were collected by centrifuging at 200 × *g* for 10 min at 4 °C.

### Histological analysis

Fresh tissues were fixed in 4% paraformaldehyde, embedded in paraffin, cut into 4-μm sections. Then the sections were used for hematoxylin-eosin (H&E) staining or Alcian blue and periodic-acid Schiff (AB-PAS) staining according to the manufacturer’s instructions. For immunohistochemistry (IHC) staining, the sections were stained with the antibodies listed in Supplementary Table S1.

### TUNEL assay

Apoptosis was detected in paraffin-embedded colon samples using the One Step TUNEL Apoptosis Assay Kit (C1086, Beyotime) according to the manufacturer’s instructions.

### ATP quantitative detection

Intestinal epithelial cells were isolated from fresh colonic tissues and then the ATP detection kit (S0026, Beyotime) was used to detect the level of ATP according to the manufacturer’s instructions.

### Analysis of intestinal permeability in mice

In vivo intestinal permeability was determined as previously described 25. Briefly, mice were starved overnight, and FITC-dextran (Sigma #FD4) was administered by oral gavage (44 mg/100 g body weight). After 4 h, mice were anesthetized and blood was collected. The concentration of FITC in serum was determined by spectrophotofluorimetry, with an excitation wave length of 485 nm and an emission wave length of 528 nm, using serially diluted FITC-dextran as standard.

### RNA extraction and quantitative PCR (qPCR)

Total RNA was extracted with TRIzol reagent (Invitrogen Life Technologies) from tissues according to the manufacturer’s instructions. Complementary DNA was synthesized using the PrimeScript RT reagent kit (Takara), and qPCR was performed in triplicates using SYBR Green PCR reagents (Takara) on an ABI Stepone Plus system (Applied Biosystems). Relative mRNA expression level was determined using the ^ΔΔ^Ct method with 18s rRNA as the internal reference. Sequences of the qPCR primers used are listed in Supplementary Table 2.

### Protein extraction and western blotting analysis

Total protein was extracted from tissues using the lysis buffer containing 50 mM Tris-HCl (pH 8.0), 150 mM NaCl, 1% NP-40, 0.5% sodium Deoxycholate and 1% SDS. After centrifugation at 13,000 rpm for 15 min, supernatants were collected for protein determinations and SDS-PAGE analysis.

### BrdU pulse-chase experiment

Proliferation rates were determined by administering mice with 80 mg/kg BrdU (bromodeoxyuridine) intraperitoneally 4 h before euthanization. Segments of mouse colons were opened longitudinally, rinsed in PBS, fixed in paraformaldehyde, and embedded in paraffin. Immunohistochemistry was used to detect BrdU-labeled cells in colons.

### Isolation of lamina propria cells

Mice were sacrificed and the colons were removed and cut into 4–5 cm pieces. The pieces were washed with ice-cold PBS and incubated in 5 ml pre-digestion solution (1X PBS containing 5 mM EDTA and 1 mM DTT) for 30 min at 37 °C with slow rotation (180 rpm) before being passed through a 40 μm cell strainer. The remaining tissues were cut into 1 mm pieces and incubated with 100 U/ml collagenase VIII and 0.8 mg/ml DNase I at 37 °C for 1 h. The supernatant was passed through a 40 μm cell strainer and cells were collected by centrifuging at 300 × *g* for 5 min at 4 °C.

### Flow cytometric analysis

Cells were isolated from mouse colons and dead cells were excluded by Fixable viability Dye eFluor 780 staining. For the analysis of surface markers, cells were stained in PBS containing 2% FBS and 2 mM EDTA. To determine cytokine expression of neutrophils, cells were stimulated with LPS (125 ng/ml) at 37 °C for 2 h. For lymphocytes, cells were stimulated with PMA and ionomycin in the presence of Golgi stop agent to detect intracellular cytokine production. After stimulation, cells were stained with antibodies against surface markers and Fixable viability Dye eFluor 780, fixed and permeabilized with Intracellular fixation buffer, following by staining with cytokine antibodies. All samples were acquired with an LSRFortessa X-20 cell analyzer (BD Bioscience) and analyzed with FlowJo software (TreeStar). Monoclonal antibodies against mouse proteins including APC-conjugated CD45, PerCP-Cy5.5-conjugated CD45, PerCP-Cy5.5-conjugated TCRβ, PE-conjugated CD19, FITC-conjugated NK1.1, BV421-conjugated CD4, PE-Cy7-conjugated CD8, BV785-conjugated CD11b, PerCP-Cy5.5-conjugated CD11c, BV650-conjugated Ly6G, PE-Cy7-conjugated Ly6C, BV510-conjugated MHC II, PE-conjugated CD64, PE-conjugated TNFα and APC-conjugated IL-1β were purchased from eBioscience, BD Bioscience or Biolegend. The antibodies were listed in Supplementary Table 1.

### RNA-seq analysis

Total RNA was extracted from WT and TKT-deficient colons. The mRNA was isolated with Oligo Magnetic Beads and randomly interrupted using divalent cation in NEB Fragmentation Buffer for cDNA synthesis. Libraries were generated using the NEBNext UltraTM RNA Library Prep Kit following the manufacturer’s instructions. Sequencing was conducted using the Illumina Hiseq XTEN platform.

### Metabolomics profiling

50 mg of samples were homogenized in precooled extractant of 70% methanol aqueous solution, followed by centrifugation at 12,000 rpm for 10 min at 4 °C. 200 μL of supernatant was subjected to LC-MS/MS analysis. 2 μL sample was injected for each analysis. A SeQuant ZIC-pHILIC column (5 µm, 2.1 × 100 mm) was used for separation at 40 °C. The mobile phase consisted of solution A (water with 5 mmol/L ammonium acetate and 5 mmol/L ammonia solution) and solution B (acetonitrile).

### Determination of inflammatory cytokines

Levels of serum IL-6 and TNFα were determined using the kits shown in Supplementary Table 1, according to the manufacturer’s instructions.

### Statistical analysis

All the replicate experiments are biological replicates, which were repeated at least three times. Data are represented as mean ± SEM. Statistical significance was assessed by two-tailed Student’s *t* test. The Kaplan–Meier survival data were analyzed by log-rank test. Analysis was made using GraphPad Prism 6.0 software. *P* < 0.05 was considered statistically significant.

## Supplementary information

Supplementary Information file

## Data Availability

All data generated or analyzed during this study are included in this published article. Further details are available from the corresponding author upon request.

## References

[1] van der Flier LG, Clevers H (2009). Stem cells, self-renewal, and differentiation in the intestinal epithelium. Annu Rev Physiol.

[2] Barker N (2014). Adult intestinal stem cells: critical drivers of epithelial homeostasis and regeneration. Nat Rev Mol Cell Biol.

[3] Peterson LW, Artis D (2014). Intestinal epithelial cells: regulators of barrier function and immune homeostasis. Nat Rev Immunol.

[4] Edelblum KL, Yan F, Yamaoka T, Polk DB (2006). Regulation of apoptosis during homeostasis and disease in the intestinal epithelium. Inflamm Bowel Dis.

[5] Algieri F, Rodriguez-Nogales A, Garrido-Mesa J, Camuesco D, Vezza T, Garrido-Mesa N (2016). Intestinal anti-inflammatory activity of calcium pyruvate in the TNBS model of rat colitis: comparison with ethyl pyruvate. Biochem Pharm.

[6] Davé SH, Tilstra JS, Matsuoka K, Li F, DeMarco RA, Beer-Stolz D (2009). Ethyl pyruvate decreases HMGB1 release and ameliorates murine colitis. J Leukoc Biol.

[7] Nielsen OH, Li Y, Johansson-Lindbom B, Coskun M (2017). Sphingosine-1-phosphate signaling in inflammatory bowel disease. Trends Mol Med.

[8] Machiels K, Joossens M, Sabino J, De Preter V, Arijs I, Eeckhaut V (2014). A decrease of the butyrate-producing species Roseburia hominis and Faecalibacterium prausnitzii defines dysbiosis in patients with ulcerative colitis. Gut.

[9] Nikolaus S, Schulte B, Al-Massad N, Thieme F, Schulte DM, Bethge J (2017). Increased tryptophan metabolism is associated with activity of inflammatory bowel diseases. Gastroenterology.

[10] Couto MR, Goncalves P, Magro F, Martel F (2020). Microbiota-derived butyrate regulates intestinal inflammation: focus on inflammatory bowel disease. Pharm Res.

[11] Sun X, Yao L, Liang H, Wang D, He Y, Wei Y, (2019). Intestinal epithelial PKM2 serves as a safeguard against experimental colitis via activating beta-catenin signaling. Mucosal Immunol.

[12] Iatsenko I, Boquete JP, Lemaitre B (2018). Microbiota-derived lactate activates production of reactive oxygen species by the intestinal NADPH oxidase Nox and shortens Drosophila lifespan. Immunity.

[13] Cheung EC, Athineos D, Lee P, Ridgway RA, Lambie W, Nixon C, (2013). TIGAR is required for efficient intestinal regeneration and tumorigenesis. Dev Cell.

[14] Ge T, Yang J, Zhou S, Wang Y, Li Y, Tong X (2020). The role of the pentose phosphate pathway in diabetes and cancer. Front Endocrinol.

[15] Patra KC, Hay N (2014). The pentose phosphate pathway and cancer. Trends Biochem Sci.

[16] Tian N, Liu Q, Li Y, Tong L, Lu Y, Zhu Y (2020). Transketolase deficiency in adipose tissues protects mice from diet-induced obesity by promoting lipolysis. Diabetes.

[17] Li M, Lu Y, Li Y, Tong L, Gu XC, Meng J (2019). Transketolase deficiency protects the liver from DNA damage by increasing levels of ribose 5-phosphate and nucleotides. Cancer Res.

[18] Iraporda C, Romanin DE, Bengoa AA, Errea AJ, Cayet D, Foligné B (2016). Local treatment with lactate prevents intestinal inflammation in the TNBS-induced Colitis model. Front Immunol.

[19] Ghishan FK, Kiela PR (2017). Vitamins and minerals in inflammatory bowel disease. Gastroenterol Clin North Am.

[20] Mańkowska-Wierzbicka D, Michalak S, Karczewski J, Dobrowolska A, Wierzbicka A, Stelmach-Mardas M (2019). Erythrocyte transketolase deficiency in patients suffering from Crohn’s disease. Eur Rev Med Pharm Sci.

[21] Bentz S, Pesch T, Wolfram L, de Vallière C, Leucht K, Fried M, (2011). Lack of transketolase-like (TKTL) 1 aggravates murine experimental colitis. Am J Physiol Gastrointest Liver Physiol.

[22] Xu ZP, Wawrousek EF, Piatigorsky J (2002). Transketolase haploinsufficiency reduces adipose tissue and female fertility in mice. Mol Cell Biol.

[23] Tsukita S, Furuse M, Itoh M (2001). Multifunctional strands in tight junctions. Nat Rev Mol Cell Biol.

[24] Slifer ZM, Blikslager AT. The integral role of tight junction proteins in the repair of injured intestinal epithelium. Int J Mol Sci. 2020;21:972. 10.3390/ijms21030972 PMC7036844 32024112

[25] Gupta J, del Barco Barrantes I, Igea A, Sakellariou S, Pateras IS, Gorgoulis VG, (2014). Dual function of p38alpha MAPK in colon cancer: suppression of colitis-associated tumor initiation but requirement for cancer cell survival. Cancer Cell.

[26] Dirisina R, Katzman RB, Goretsky T, Managlia E, Mittal N, Williams DB, (2011). p53 and PUMA independently regulate apoptosis of intestinal epithelial cells in patients and mice with Colitis. Gastroenterology.

[27] Kuo WT, Shen L, Zuo L, Shashikanth N, Ong M, Wu L, (2019). Inflammation-induced Occludin downregulation limits epithelial apoptosis by suppressing caspase-3 expression. Gastroenterology.

[28] Nenci A, Becker C, Wullaert A, Gareus R, van Loo G, Danese S (2007). Epithelial NEMO links innate immunity to chronic intestinal inflammation. Nature.

[29] Cosentino K, Garcia-Saez AJ (2017). Bax and Bak pores: are we closing the circle?. Trends Cell Biol.

[30] Warren CFA, Wong-Brown MW, Bowden NA (2019). BCL-2 family isoforms in apoptosis and cancer. Cell Death Dis.

[31] Eissa N, Hussein H, Diarra A, Elgazzar O, Gounni AS, Bernstein CN, (2019). Semaphorin 3E regulates apoptosis in the intestinal epithelium during the development of colitis. Biochem Pharm.

[32] Gunther C, Buchen B, Neurath MF, Becker C (2014). Regulation and pathophysiological role of epithelial turnover in the gut. Semin Cell Dev Biol.

[33] Nita M, Grzybowski A (2016). The role of the reactive oxygen species and oxidative stress in the pathomechanism of the age-related ocular diseases and other pathologies of the anterior and posterior eye segments in adults. Oxid Med Cell Longev.

[34] Richter C, Schweizer M, Cossarizza A, Franceschi C (1996). Control of apoptosis by the cellular ATP level. FEBS Lett.

[35] Mulukutla BC, Yongky A, Le T, Mashek DG, Hu WS (2016). Regulation of glucose metabolism - a perspective from cell bioprocessing. Trends Biotechnol.

[36] Xavier RJ, Podolsky DK (2007). Unravelling the pathogenesis of inflammatory bowel disease. Nature.

[37] Qiu W, Wu B, Wang X, Buchanan ME, Regueiro MD, Hartman DJ (2011). PUMA-mediated intestinal epithelial apoptosis contributes to ulcerative colitis in humans and mice. J Clin Invest.

[38] Circu ML, Aw TY (2010). Reactive oxygen species, cellular redox systems, and apoptosis. Free Radic Biol Med.

[39] Aviello G, Knaus UG (2018). NADPH oxidases and ROS signaling in the gastrointestinal tract. Mucosal Immunol.

[40] Eguchi Y, Shimizu S, Tsujimoto Y (1997). Intracellular ATP levels determine cell death fate by apoptosis or necrosis. Cancer Res.

[41] Skulachev VP (2006). Bioenergetic aspects of apoptosis, necrosis and mitoptosis. Apoptosis.

[42] Leist M, Single B, Castoldi AF, Kühnle S, Nicotera P (1997). Intracellular adenosine triphosphate (ATP) concentration: a switch in the decision between apoptosis and necrosis. J Exp Med.

[43] Moley KH, Mueckler MM (2000). Glucose transport and apoptosis. Apoptosis.

[44] Liu X, Lu J, Liu Z, Zhao J, Sun H, Wu N (2018). Intestinal epithelial cell-derived LKB1 suppresses colitogenic microbiota. J Immunol.

