# Supplementary material for: TKT maintains intestinal ATP production and inhibits apoptosis-induced colitis
Source: Cell Death Dis. 2021 Sep 17;12(10):853. doi: 10.1038/s41419-021-04142-4 (PMC8448773; doi:10.1038/s41419-021-04142-4)
Supplement: Supplementary file 1 — Supplementary Information file [file 41419_2021_4142_MOESM1_ESM.pdf]

Figure S1

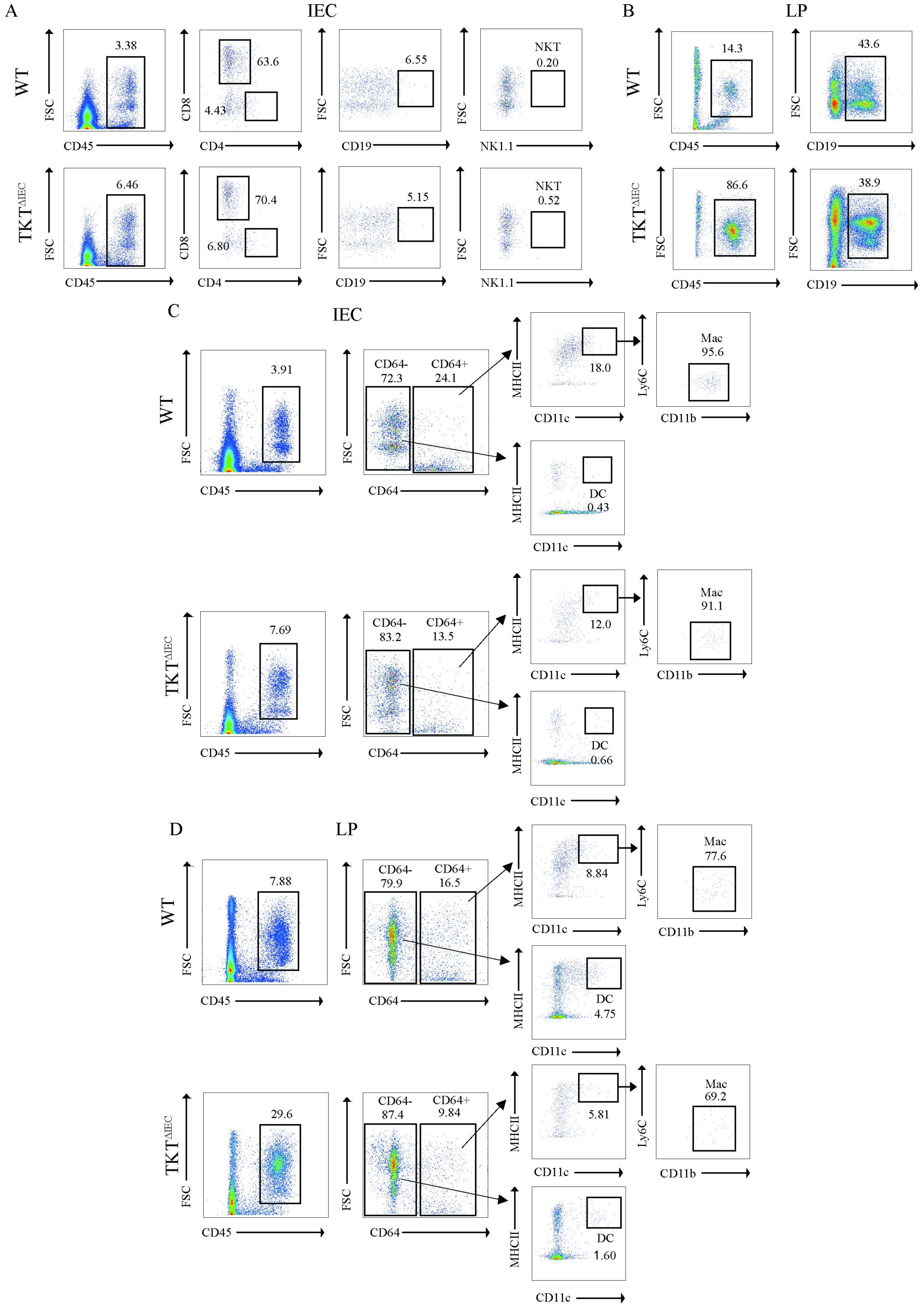

Fig. S1 Colon-infiltrating T cells slightly increase in TKT$^{\Delta IEC}$ mice whereas B cells, macrophages and dendritic cells remain unchanged between WT and TKT$^{\Delta IEC}$

mice. (A) Representative flow cytometric analysis of the population of $CD8^+$ T cells, $CD4^+$ T cells, B cells and NKT cells in the IEC of colons from 6-week-old WT and $TKT^{\Delta IEC}$ mice (n=3). (B) Representative flow cytometric analysis of the population of B cells in the LP of colons from 6-week-old WT and $TKT^{\Delta IEC}$ mice (n=3). (C) Representative flow cytometric analysis of the population of macrophages and dendritic cells in the IEC of colons from 6-week-old WT and $TKT^{\Delta IEC}$ mice (n=3). (D) Representative flow cytometric analysis of the population of macrophages and dendritic cells in the LP of colons from 6-week-old WT and $TKT^{\Delta IEC}$ mice (n=3).

Fig. S2

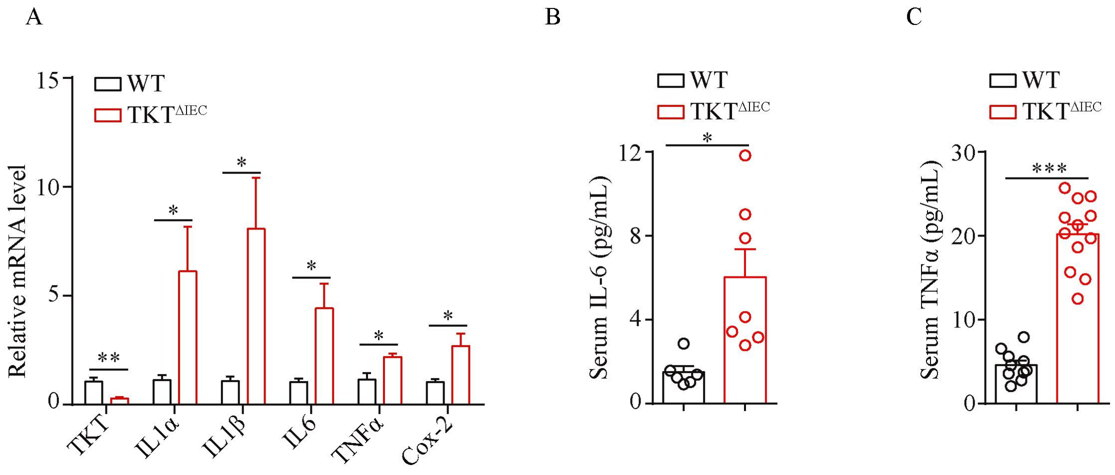

Fig. S2 Loss of TKT in intestinal epithelium induces intestinal inflammation. (A) QPCR analysis of colonic inflammatory gene expression of 8-week-old WT and TKT$^{\Delta IEC}$ mice (n=5). (B) Serum levels of IL-6 of 4-week-old WT and TKT$^{\Delta IEC}$ mice. (C) Serum levels of TNFα of 4-week-old WT and TKT$^{\Delta IEC}$ mice.

Supplementary Table 1

Reagent	Company	CAT#
ATP Assay Kit	Beyotime	S0026
One Step TUNEL Apoptosis Assay Kit	Beyotime	C1086
Mouse IL-6 Quantikine ELISA Kit	R&D Systems	M6000B
Mouse TNF-alpha Quantikine ELISA Kit	R&D Systems	MTA00B
NADP/NADPH Quantitation Kit	Sigma	MAK038
HRP-conjugated α-tubulin	Proteintech	66031
Anti-β-Actin	Proteintech	66009
Anti-Cleaved caspase-3	Cell Signaling Technology	9661
Anti-Bax	Cell Signaling Technology	2772
Anti-Ki67	Cell Signaling Technology	12202
Anti-CD45	BD Biosciences	550539
Anti-BrdU	Cell Signaling Technology	5292
Anti-CD45.2	eBioscience	17-0454-82
Anti-IL1β	eBioscience	17-7114-80
Anti-CD19	eBioscience	12-0193-81
Anti-TCRβ	BD Biosciences	560657
Anti-CD4	BD Biosciences	562891
Anti-CD8	eBioscience	25-0081-82
Anti-NK1.1	eBioscience	11-5941-85
Anti-TNFα	eBioscience	12-7321-82

Anti-CD45.2	eBioscience	45-0451-82
Anti-CD11b	Biolegend	101243
Anti-CD11c	eBioscience	45-0114-82
Anti-Ly6G	Biolegend	127641
Anti-Ly6C	Biolegend	128017
Anti-CD64	Biolegend	139304
Anti- MHC Ⅱ	Biolegend	107636
Anti-ZO-1	Proteintech	21773-1-AP
Anti-Occludin	Proteintech	13409-1-AP
Anti-Caspase3	Cell Signaling Technology	9662

Supplementary Table 2

Primers for qPCR		
Gene name	Forward primer	Reverse primer
18s rRNA	TTGACTCAACACGGGAAACC	AGACAAATCGCTCCACCAAC
TKT	ATGTCCACCGTCTTTTACCC	TCACTTGGTCATCCTTGCTC
IL1α	GAGAGCCGGGTGACAGTATC	TGACAAACTTCTGCCTGACG
IL1β	CCCCAACTGGTACATCA	AGAATGTGCCATGGTTTC
IL6	AGTTGCCTTCTTGGGACTGA	CAGAATTGCCATTGCACAAC
TNFα	CGTCAGCCGATTTGCTATCT	CGGACTCCGCAAAGTCTAAG
Cox-2	CAGCACTTCACCCATCAGTTT	GGCGCAGTTTATGTTGTCTG
Muc2	GACCTCCACCCCTACAAACA	TTGTTCCAGGGGATGATGCT

Alpi	ACCCAGCAGTAACTCACCTC	CCTCAGTCAGTGCCAGGTAA
Lysozyme	ATGGCTACCGTGGTGTCAAG	CGGTCTCCACGGTTGTAGTT
ChgA	CGATCCAGAAAGATGATGGTC	CGGAAGCCTCTGTCTTTCC
ZO-1	GCCGCTAAGAGCACAGCAA	GCCCTCCTTTTAACACATCAGA
ZO-2	ACTCCAGTCCCTATTCCTGAG	GCTATTTCGATCCTCGCATTC
Occludin	TTGAAAGTCCACCTCCTTACAGA	CCGGATAAAAAGAGTACGCTGG
Bax	CTGAGCTGACCTTGGAGC	GACTCCAGCCACAAAGATG
Bcl-2	TGTGGTCCATCTGACCCTCC	ACATCTCCCTGTTGACGCTCT
Ki67	GTGCTGACCCTGATGGGGAAGG	GCTCTTGCCCTGCCTGACACC
Acadsb	TTATGCATCTGAGGTCGCTGG	GGTGTTCAGCTGGATGTTGG
Cpt1α	GTGGGAGCGACTCTTCAATAC	ATCTGCTGCTCCAGCTCAC
Ehhadh	GGCGCAGGATACCTTGAGAA	TGATTACAGTTGGACTGATGGCA
Fabp2	GGAAAGTAGACCGGAACGAG	ACACCGAGCTCAAACACAAC
Hadh	GGTCCGTTTGAGCTTCTTG	ATCCTTCTCCGGTCTTCTTG
Mut	CCCAAACACTGACCGTTCTCAT	GCTTCAGGTAATGGGGCGATA
Slc27a2	GTCTCTGCTGCACTGCTTTC	TGACTTCAGACCTCCACGAC
BCAT2	TTCATTCGTCAGAGCCTGGATA	ACTACTCCAGGCAAGATGACGC
Bckdha	AACCAGCCCTTCCTCATTGA	GGCCTTCTCCTGTTCCTCAT
Bckdhb	TTCCATGGCCCAAGAAAAGC	AAGATGTGAGGAAACGGGGT
Dbt	CAAAGACCGAACAGAGCCAG	ACGGAGGCATTGAGGATAGG
